# Dr. Robert Hugh McKay Dawbarn

**Published:** 1915-09

**Authors:** 


**Dr. Robert Hugh McKay Dawbarn,** P. & S. 1881, a well known surgeon of N. Y. City, died July 18, aged 66.

